# Non coding RNAs in aortic aneurysmal disease

**DOI:** 10.3389/fgene.2015.00125

**Published:** 2015-04-01

**Authors:** Aparna Duggirala, Francesca Delogu, Timothy G. Angelini, Tanya Smith, Massimo Caputo, Cha Rajakaruna, Costanza Emanueli

**Affiliations:** ^1^Bristol Heart Institute, School of Clinical Sciences, University of BristolBristol, UK; ^2^Royal Surrey County HospitalGuildford, UK; ^3^Rush Centre for Congenital and Structural Heart Disease, Rush University Medical CentreChicago, IL, USA

**Keywords:** microRNAs, long non-coding RNAs, aneurysms, vascular cells, therapeutic targets, biomarkers

## Abstract

An aneurysm is a local dilatation of a vessel wall which is >50% its original diameter. Within the spectrum of cardiovascular diseases, aortic aneurysms are among the most challenging to treat. Most patients present acutely after aneurysm rupture or dissection from a previous asymptomatic condition and are managed by open surgical or endovascular repair. In addition, patients may harbor concurrent disease contraindicating surgical intervention. Collectively, these factors have driven the search for alternative methods of identifying, monitoring and treating aortic aneurisms using less invasive approaches. Non-coding RNA (ncRNAs) are emerging as new fundamental regulators of gene expression. The small microRNAs have opened the field of ncRNAs capturing the attention of basic and clinical scientists for their potential to become new therapeutic targets and clinical biomarkers for aortic aneurysm. More recently, long ncRNAs (lncRNAs) have started to be actively investigated, leading to first exciting reports, which further suggest their important and yet largely unexplored contribution to vascular physiology and disease. This review introduces the different ncRNA types and focus at ncRNA roles in aorta aneurysms. We discuss the potential of therapeutic interventions targeting ncRNAs and we describe the research models allowing for mechanistic studies and clinical translation attempts for controlling aneurysm progression. Furthermore, we discuss the potential role of microRNAs and lncRNAs as clinical biomarkers.

## Introduction

**Figure 1:**
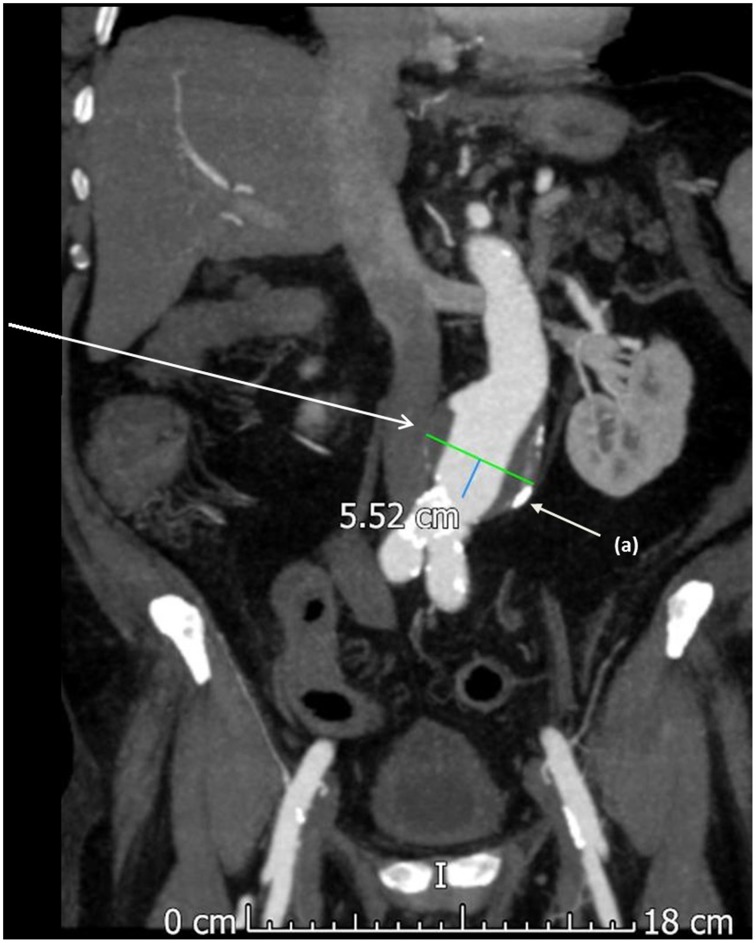
**A computed tomography (CT) scan with contrast showing an abdominal aortic aneurysm**. This aneurysm was asymptomatic and found during routine surveillance scanning of a 67 year old male. Calcified atherosclerotic plaque is noted on the aneurysm wall and in the distal aorta (a).

**Figure 2:**
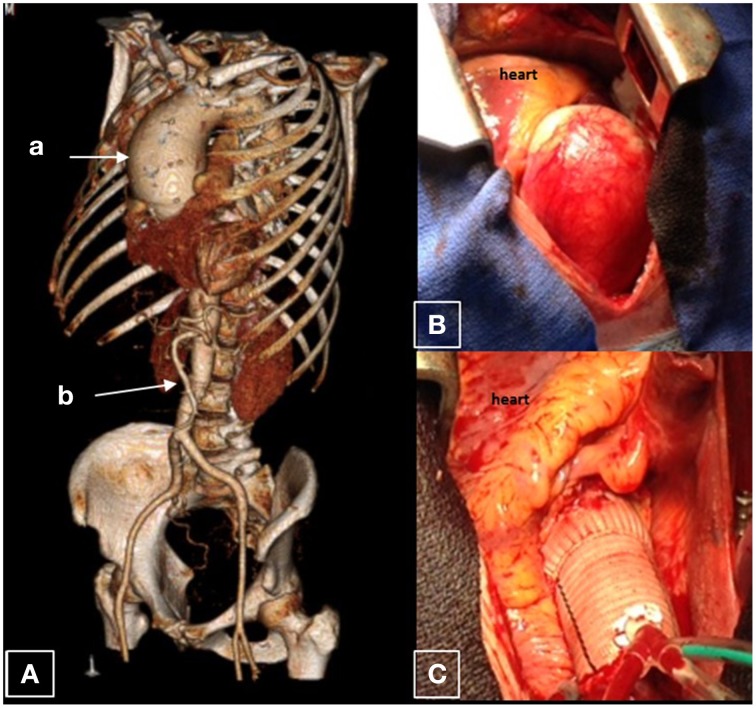
**Ascending aortic aneurysm**. This is an aorta of a 37 year old male who was found to have a 7.7 cm saccular ascending aortic aneurysm associated with a leaking bicuspid aortic valve. The remainder of the aorta is normal caliber. **(A)** CT reconstruction of the whole aorta with ascending aortic aneurysm (a), with a normal caliber abdominal aorta (b). **(B)** Surgical view of this aneurysm; **(C)** Dacron graft after surgical replacement.

**Figure 3:**
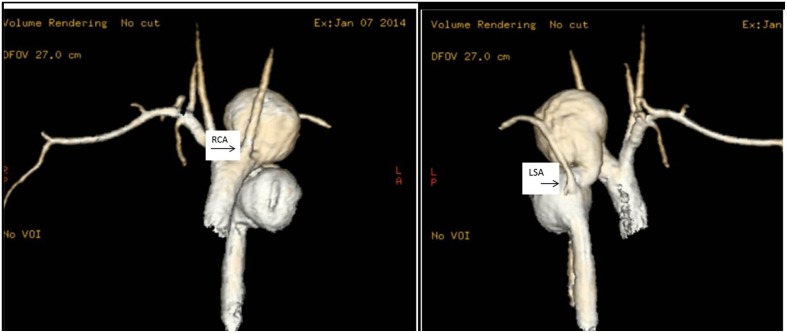
**Thoracic aorta aneurysms**. MRI scans of the ascending aorta in a 17 year old girl with multiple thoracic aortic aneurysms. In (i), it is clearly visible a large saccular aneurysm of the aortic arch just distal to the right carotid artery (RCA) and a second large saccular aneurysm in the descending aorta. In (ii), the MRI scan shows the ascending and descending aorta. The left subclavian artery (LSA) comes off the descending aorta in between two large saccular aneurysms.

Arterial aneurysms (AAs) are asymmetrical dilatations of arterial wall, most commonly found in the infra-renal abdominal aorta (see Figure 1). Lesions develop when intraluminal pressures exceed the capacity of the arterial wall which has developed a localized weakness. It is estimated that abdominal aortic aneurysms (AAA) account for approximately 2–4% of all deaths in men aged 65 and over (Clouse et al., 1998; Booher and Eagle, 2011). Thoracic aortic aneurysms (TAAs) (Figures 2, 3) represent the most lethal site for a dilatation. They may remain asymptomatic for years and manifest as an acute rupture or dissection which can be fatal if left untreated. The prevalence of thoracic aneurysms in the United States is estimated at 10.4 per 100,000 people (Clouse et al., 1998). TAAs is a silent disease and 95% of cases present acutely from previously asymptomatic patients (Clouse et al., 1998). Thoracic aortic aneurysms are classified according to the segment of the dilatation. This anatomical classification is important because the etiology, natural history and treatment may differ for each segment.

The ascending thoracic aorta and the descending/abdominal aortas have different embryological aetiologies, pathological features and their evolution progresses differently (better described below—Section Mechanisms Underlying Abdominal Aortic Aneurysms). Aneurysms are much more common in the abdominal aorta than in the ascending aorta. Classically, atherosclerosis has been considered the underlying cause of abdominal aortic aneurysms (Lederle et al., 1997; Guo et al., 2001). Known cardiovascular risk factors mostly associated with AAA include smoking, increasing age, hypertension, hyperlipidaemia which predispose to atherosclerosis (Lederle et al., 1997; Coady et al., 1999). On the other hand, atherosclerosis is infrequently implicated in ascending thoracic aortic aneurysm development. Ascending thoracic aneurysms are associated with hypertension, connective tissue disease, bicuspid aortic valves, familial thoracic aneurysm syndrome, (Coady et al., 1999; Guo et al., 2001; Nuenninghoff et al., 2003; Demers et al., 2004).

This review focuses on non-coding (nc) RNAs as possible new mechanisms of diagnosis and monitoring of aneurysmal disease and novel therapeutic enabling to control the progression to dissection and rupture. The recent advancement in DNA and RNA sequencing techniques have led to the understanding that around 98% of the human genome contains regions of DNA which code for RNA molecules unable to produce proteins and hence termed ncRNAs. Far from being “junk RNA,” many ncRNAs are proving to be very powerful regulators of gene expression, acting at both transcriptional and post-transcriptional level. ncRNAs are arbitrarily defined accordingly to their size, as small ncRNAs and long ncRNAs (lncRNAs). MicroRNAs (miRNAs, miRs) are the most popular small ncRNAs. Here, we will review miRNA and lncRNAs for their regulatory roles in vascular function, with special attention to their contribution to VSMC function and vascular degenerative disease. Finally, we will discuss the potential of ncRNAs as new therapeutic targets and clinical biomarkers to be employed in patients with aneurysms at different stage of evolution.

## Non-coding RNAs

About 2% of the eukaryotic genome contains protein coding genes and the remaining DNA was previously supposed to be “junk DNA,” i.e., DNA devoid of any functional importance. In fact, large-scale analyses of mammalian transcriptomes have identified 60% of RNA molecules that are not translated into protein. However, the use of new sequencing technologies has identified that most of the genome is transcribed, producing a heterogeneous population of “noncoding RNA” (ncRNAs), which do not hold information for protein translation. Although ncRNAs do not code for proteins, they play different roles in regulating gene expression, thus impacting on cell functions. According to their size, ncRNA are classified as “small ncRNA” (20–200 nucleotides in their mature forms) and “long ncRNA” (more than 200nt and up to 100 kilobases). According to their function, ncRNAs can also be classified as “housekeeping” ncRNAs, which are constitutively expressed and crucial for normal function and cellular viability. These include tRNA, rRNA, SnoRNA. By contrast, the “regulatory” ncRNAs, which are important for the transcriptional regulation of genes. In recent years, several ncRNAs have been found to regulate vascular pathologies, including aortic aneurysm (thoracic and abdominal) disease.

### MicroRNAs

MiRNAs are post transcriptional regulators of gene expression. They influence the stability and translation of messenger RNAs (mRNAs). MiRNA are encoded (often as polycistronic “clusters”) in intergenic region of protein coding genes or by miRNA genes. MiRNA genes are transcribed by RNA pol lI as capped and polyadenylated primary miRNA transcripts (pri-mRNA). The pri mRNA is then processed in two independent steps by the enzymes Drosha and DICER. Dorsha/DGCR8 cleaves the pri-miRNA transcript to a 60–100 nucleotide long hairpin loop which is transported to the cytoplasm where it is further processed to mature miRNA, a single stranded of ~22 nucleotides. This can be incorporated into the RNA-induced silencing complex (RISC) and mediates gene silencing by affecting the integrity and/or the protein synthesis capacity of the targeted mRNAs. A miRNA recognize its mRNA targets mRNAs by the semi complementarity between its “seed sequence” region and one or more “miRNA binding sites,” usually located in the 3′ untranslated region (3′ UTR) of the mRNAs(Gregory et al., 2006).

### Long non coding RNAs

The RNA transcripts that are longer than 200 nucleotides which are polyadenylated and are devoid of open reading frames are defined as lncRNAs. The functional annotation of mammalian cDNA (FANTOM3) project identified 35,000 of such non coding transcripts (Amaral et al., 2011). LncRNA are located and transcribed with the intergenic region of genome by the RNA PolI enzyme or in some cases by RNA pol III. The majority are transcribed as complex, interlaced networks of overlapping sense and antisense transcripts. On the basis of their genomic loci, long ncRNA (lncRNAs) can be classified onto: (1) Sense lncRNAs, transcribed from a locus overlapping with protein coding gene; (2) Antisense lncRNAs loci, that overlap with the antisense strand of the protein coding gene; (3) Bidirectional lncRNAs, located on the antisense strand of the protein coding gene; (4) Intronic lncRNAs, transcribed from introns to protein coding genes; (5) Long intergenic ncRNAs (lincRNAs), that are not in the proximity of protein coding gene. LncRNAs display high organ and cell specificity and are not well conserved across species. They can be located in the nucleus or in the cytosol and exert actions *in cis* and *in trans*. LncRNAs can regulate different aspects of transcription by playing different roles, including acting on transcription factors (TFs), RNA polII, mRNA processing, splicing and the epigenetic machinery (Kapranov et al., 2007; Broadbent et al., 2008; Mohammad et al., 2008). Additionally, lncRNAs can exert a post-transcriptional regulation of protein coding genes, for example by acting as antisenses inhibitors of mRNAs (Kornienko et al., 2013).

Exploratory studies performed on the lncRNA in the cardiovascular setting have so far identified a few lncRNA associated with cardiovascular diseases. These include MALAT1 (metastasis associated lung adenocarcinoma transcript 1), LIPCAR (lincRNA predicting cardiac remodeling), CARL (cardiac apoptosis related lncRNA), MiAT (myocardial infarction–associated transcript), HCG22 (HLA complex group 22), Brave heart, CHRF (cardiac hypotrophy factor), ANRIL (antisense ncRNA in the INK4 locus), and HIF1A-AS1 (antisense hypoxia inducible factor 1 alpha antisense RNA), KCNQ1OT (KCNQ1 overlapping transcript), and SENCR (smooth muscle and endothelial cell-enriched migration/differentiation-associated lncRNA). Specific studies looking at lncRNAs in aortic aneurysm are still lacking (Skroblin and Mayr, 2014). Because of the high number of lncRNAs, research based on predefined lncRNA candidate is not ideal and next generation sequencing methods will be useful in exploring lncRNA which can be functionally related with aortic aneurysms and it might support the identification of lncRNAs to be investigated as biomarkers for diagnosing sporadic aortic aneurysms and to follow up aneurysm evolution.

## Cellular mechanisms and signaling pathways underpinning aneurysm formation and evolution

The currently available pharmacological approaches are unable to prevent aneurism progression. Although there is some success demonstrated with Angiotensin II (Ang II) receptor blockers in non-pathological aneurysms in Marfan's patients (Attenhofer Jost et al., 2014), this therapeutic approach is not proven to be effective enough to substitute the need for surgical therapy. This lack of effective therapies suggests the need for a better understanding the molecular mechanisms underpinning aneurysm pathology and progression. New mechanistic insight should provide better targets for therapeutic intervention. In this section, we'll describe the pathways and cellular mechanisms underlying aneurysm formation and evolution.

### Mechanisms underlying abdominal aortic aneurysms

Abdominal aortic aneurysms have been linked to atherosclerosis and the pathophysiology of this aneurysm has been well studied (Guo et al., 2006; Shimizu et al., 2006). Histological analyses of human AAA samples revealed the three pathological hall marks of abdominal aorta aneurysms: leukocyte infiltration, degradation of extra cellular matrix (ECM) and VSMC depletion (Guo et al., 2006). Moreover, work on animal models (described below) confirmed this data and further indicated that abdominal aneurysm development involves local inflammatory responses leading to infiltration of macrophages, neutrophils, mast cells and T and B lymphocytes (Sun et al., 2007). The prominent inflammatory cells present in aortic *tunica media* and *adventitia* are macrophages. Macrophage infiltration is critical for abdominal aneurysm development, but its contribution has not been defined. CCR2 (C-C chemokine receptor 2) and myeloid differentiation factor are important for macrophage mediated response to inflammation. CCR2 deficiency in mouse models attenuates the severity of the induced aneurysm (Daugherty et al., 2010). Neutrophils are also present in experimental and human AAA tissue. L selectin adhesion molecule mediates the trafficking of neutrophils from plasma to the aortic wall and L selectin attenuates abdominal aneurysm formation in mouse models (Ishibashi et al., 2004).

The plasma concentration of certain cytokines, such as tumor necrosis factor alpha (TNF-α), increases in patients with abdominal aneurysm. However, TNF alpha receptor deficiency has no significant effect on Ang II- induced AAA formation in LDL receptor deficient atherosclerotic mice. Another cytokine that plays a critical role is transforming growth factor beta (TGF-β). Systemic blockade of TGF-β activity was found to increase Ang II-induced AAA (Wang et al., 2010).

The drug cyclosporin induces TGF- β expression and its administration stops aneurysm formation in the rat elastase and mouse calcium chloride models of AAA (Dai et al., 2011). Interleukin 1β, IL6, IL17, and IL23 are increased in AAA in human tissue as compared with healthy tissue (Szekanecz et al., 1994). AAA formation and progression is also mediated by various proteases, which degrade elastin and collagen present in the aortic wall. Matrix metalloproteinase-9 (MMP-9) is a zinc endopeptidase contributing to calcium chloride-induced aneurysm development in mice (Tanaka et al., 2009). C-Jun N terminal kinase (JNK) was found increased in abdominal aneurysms in murine models. Moreover, a JNK specific inhibitor prevented the aneurysms formation in these models (Yoshimura et al., 2005).

### Thoracic aorta aneurysm pathology and mechanisms

Thoracic aortic aneurysm (TAA) is caused by proteoglycan accumulation, elastic fiber fragmentation, focal or diffuse VSMC degradation and loss. The pathological changes can result in excessive degradation of ECM components, leading to the loss of mechanical strength and integrity, aortic dilation, dissection, or rupture (El-Hamamsy and Yacoub, 2009). Histopathological findings of the aortic wall in patients with ascending aorta aneurysm reveal the presence of cystic medial necrosis or medial degeneration. The aneurysm is characterized by disruption of the lamellar organization of elastic fibers, cyst like lesions within the tunica media, pooling of proteoglycans, elastin and collagen fiber fragmentation and coagulative necrosis of VSMCs (El-Hamamsy and Yacoub, 2009). The NOTCH pathway is activated in fibroblasts and deregulated in VSMCs of patients with thoracic aneurysm. However, the Notch signaling is downregulated in the ascending aorta of patients with bicuspid aortic valve (BAV) and in abdominal aneurysms. Genetic variation in the *NOTCH1* gene appears to confer susceptibility to ascending aortic aneurysm formation in patients with BAV (McKellar et al., 2007). More research is needed to clarify the role of the NOTCH pathway in aneurysms and to elucidate the possibility of targeting it therapeutically.

## NcRNAs in aneurysms

NcRNAs have become a subject of great interest for vascular biology and medicine. The scientists interested in aneurysm disease can combine cell and animal models and analyses on clinical samples for their ncRNA studies. Below, we report on the current knowledge and discuss the importance of research model optimization for future research.

Table 1 summarized the miRNAs currently known to be functionally involved and/or expressionally deregulated in AAs. Table 2 summarized the lncRNAs that have been shown to be involved with Vbiology and proposes their link to AA.

**Table 1:** **microRNAs which could be involved with aneurysmal disease**.

**MiRNA**	**Up/down regulation**	**Target genes**	**Function regulated**	**Expression sites**	**References**
miR143/145	Down	Klf4, myocardin, Elk-1,	Direct VSCM differentiation, repress VSCM proliferation	Murine cardiac progenitors Mice VSMCs	Cordes et al., 2009; Elia et al., 2009
miR29	up	ECM protein encoding genes (COL1A1, COL1A2, COL3A1, FBN1, ELN, MCL1, MMP2, MMP9)	Regulate ECM	Human aortic SMCs Murine models of experimental AAA	Chen et al., 2012; Maegdefessel et al., 2012
miR 205	up	TIMP3, RECK	Regulate ECM	Murine model of AAA	Kim et al., 2014
miR 26a	down	SMAD-1	Promotes vascular SMC proliferation while inhibitin g cellular differentiation and apoptosis, and alters TGF-β pathway signaling	Human aortic SMCs	Leeper et al., 2011
miR 21	up	PTEN	Modulate proliferation and apoptosis of VSMCs	Murine models of experimental AAA	Maegdefessel et al., 2012
miR 195	up	ELN, different COL isoforms, MMP2, MMP9	Regulate ECM	Mouse aorta SMCs	Zampetaki et al., 2014
miR 221/222	up	p27, p57, c-Kit	pro-proliferative, pro-migration, and anti-apoptotic effects, Promote a synthetic phenotype in VSMCs	Human aortic SMCs Aorta SMCs and ECs from rats	Davis et al., 2009; Liu et al., 2012

*AAA, abdominal aortic aneurysm; c-kit, COL1A1, collagen type 1 alpha 1; EC, endothelial cell; ELK1, ETS domain-containing protein; ELN, elastin; FBN, fibrillin; KLF4, Kruppel-like factor 4; MCL1, myeloid cell leukemia 1; MMP, matrix metalloproteinase; PTEN, phosphatase and tensin homolog; SMAD-1, mothers against decapentaplegic homolog 1; RECK, reversion-inducing-cysteine-rich protein with kazal motifs; SMC, smooth muscle cell; TIMP3, metalloproteinase inhibitor 3; VSMC, vascular smooth muscle cell*.

**Table 2:** **Long noncoding RNAs studied in vascular smooth muscle cells**.

**lncRNA**	**Class**	**Function regulated**	**References**
ANRIL	NAT	Influences VSMC function by regulating CDKN2A, CDKN2B, DAB2IP, LRP1, LRPR, CNTN3 expression	Boucher et al., 2003; Elmore et al., 2009; Gretarsdottir et al., 2010; Bown et al., 2011; Congrains et al., 2012
SENCR	Antisense	Inhibitis of VSMC migration	Bell et al., 2014
LncAng362	Antisense	Reduces VSMC proliferation	Leung et al., 2013
HIF1A-AS1	Antisense	Pro-apoptotic and anti-proliferative effect on VSMCs	Wang et al., 2014

*ANRIL, antisense non-coding RNA in the INK4 locus; SENCR, smooth muscle and endothelial cell-enriched migration/differentiation-associated lncRNA; HIF1A-AS1, antisense hypoxia inducible factor 1 alpha anti sense RNA; NAT, natural antisense transcript; LRP1, low density lipoprotein receptor-related protein 1; LRPR, low density lipoprotein receptor; CDKN, cyclin-dependent kinase inhibitors; DAB2IP, DAB2 interacting protein; CNTN3, contactin-3; VSMC, vascular smooth muscle cells*.

### Vascular cell biology

Cell culture experiments on human aortic VSMCs isolated from clinical tissue samples suggest the role of miRNAs in aortic aneurysm development. In this article we present the miRNAs studied in VSMCs. Similar studies on lncRNAs are still lacking. However, we suggest new roles for these ncRNA molecules based on the results of some pioneers studies in non-aneurysmal VSMCs (*vide infra* at LncRNAs).

#### miRNAs

The VSMC phenotype and behavior is strongly regulated by miRNAs. The phenotype of VSMCs can change between a contractile to a synthetic state and vice versa, and aortic aneurysm can be associated with a reduction of contractile VSMC number. Knockout of Dicer impairs miRNA biogenesis. In VSMCs, this induces embryonic lethality, showing abnormal development of vascular vessels. These findings suggest that miRNAs play important roles in proliferation, migration and phenotypic transformation of VSMCs. MiRNAs are additionally involved in VSMC biology and behavior, acting on cell apoptosis proliferation, and migration through modulating the expression of vasoactive molecules, cytokines, MMPs and growth factors.

The miR-29 family of miRs contains three members (miR-29a, miR-29b, and miR-29c) that are encoded by two separate *loci*, giving rise to bi-cistronic precursor miRs (miR-29a/b1 and miR-29b2/c). This family targets numerous gene transcripts that encode ECM proteins involved in fibrotic responses, including several collagen isoforms (e.g., COL1A1, COL1A2, COL3A1), fibrillin-1, and elastin (ELN) (Boon et al., 2011).

Over-expression of miR-21 increases VSMC survival and proliferation (Song et al., 2012). By contrast, miR-133 reduces proliferation and migration of VSMC through repressing transcription factor Sp-1 (Torella et al., 2011). MiR-133 is highly expressed in the healthy vasculature, especially in VSMCs. It is anti-proliferative and upregulated when VSMCs are quiescent. Overexpression of miR-133 reduced VSMC proliferation *in vitro*; while anti-miR-133 increased VSMC proliferation (Torella et al., 2011). *In vitro* studies showed that ERK1/2 activation is partly responsible for miR-133 downregulation when VSMCs are primed for the phenotypic switch (Zhan et al., 2003; Torella et al., 2011).

MiR-143/145 have been extensively studied in vascular pathology. This cluster alters the SMC phenotypic switch and its expression is decreased by acute and chronic vascular stress. The two miRNAs cooperate in the regulation of VSMC fate and plasticity by targeting several transcription factors, such as KLF4, KLF5 myocardin and Elk-1 (Boettger et al., 2009). A study of Cordes et al. (2009) demonstrates the role of the miR-143/145 cluster in VSMC differentiation and the repression of their proliferation. Zampetaki *at al* focused on miR-195 in aortic aneurysmal disease, performing both *in vitro* and *in vivo* studies (Zampetaki et al., 2014). *In vitro* experiments on murine VSMCs confirmed the involvement of miR-195 in ECM homeostasis regulation. Proteomic analyses on the conditioned medium from VSMCs transfected with either pre-miR-195 or anti-miR-195 showed that miR-195 expression is reversely correlated with elastin, a direct target of this miRNA (Zampetaki et al., 2014). Elastin is fundamental for the maintenance of aortic mechanical integrity, allowing the vessel to resume its shape after stretching or contracting. Low elastin levels are typical of aneurysmal diseases. Recently, Kim et al. showed miR-205 is upregulated in abdominal aortic endothelial cells (ECs) and miR-205 stimulates MMP activity by targeting two endogenous MMP inhibitors, known as tissue inhibitor 3 of MMP (TIMP3) and reversion-inducing cysteine-rich protein with kazal motifs (RECK) (Kim et al., 2014).

The role or miR-26a has been studied by Leeper and collaborators, working on human aortic SMCs. They performed several assays to assess how transfection with either anti-miR or pre-miR affected VSMCs behavior. The decrease in miR-26a levels was associated with a reduction in VSMCs proliferation and migration, and a significant increase of H_2_O_2_-induced apoptosis, in comparison to control cells. They demonstrate the targeted action of this miR on SMAD-1 protein in VSMCs, and thus the effects on TGB-β pathway. All these effects could be fundamental in AA development (Leeper et al., 2011).

MiR-221/222 seems to have a cell specific effect in blood vessels. In a study by Liu et al., SMCs and ECs were isolated from murine aorta samples. The effects of miR-221/222 downregulation and upregulation were analyzed on each cell type studying their proliferation, migration and apoptosis. These miRNAs resulted to have a pro-proliferative, pro-migration and anti-apoptotic effect on VSMCs, while an anti-proliferative, anti-migration and pro-apoptotic effect on ECs. The action of miR-221/222 are mediated by directly suppressing c-kit and cyclin-dependent kinase inhibitors p27Kip1 and p57Kip2, three proteins involved in key processes as cell differentiation, proliferation, migration and apoptosis (Liu et al., 2012). miR-221/222 is up-regulated, while miR-133, miR-143/145, and miR-663 are down-regulated in response to PDGF treatment on VSMCs (Liu et al., 2012). MiR-221/222 is overexpressed in injured vascular wall SMCs in rat carotid arteries after angioplasty. Specific knockdown of miR-221 and miR-222 resulted in decreased VSMC proliferation *in vitro*. Knockdown also suppressed VSMC proliferation *in vivo* and decreased neointimal lesion formation after angioplasty in rat carotid arteries (Liu et al., 2010).

#### LncRNAs

As aforementioned, here we are reviewing the actions of a few lncRNAs in vascular cell biology at large since specific studies on lncRNAs in aneurysmal VSMCs are still lacking. We are confident that this gap will be soon filled by the pioneer researchers who have started lncRNA research programs in vascular medicine.

LncRNAs regulate gene expression, including chromatin remodeling, mRNA transcription and processing, and post-transcriptional pathways. It is important to explore whether lncRNAs could be involved in vascular disease through these mechanisms. Wang et al. showed that HIF1A-AS1 mediates the pro-apoptosis and anti-proliferative responses induced by *BRG1* gene (Brahma-related gene 1) in VSMCs (Wang et al., 2014). They also showed that BRG1 expression is significantly increased in the aortic media of TAA patients compared to the normal group, implying that BRG1 may play a role in development and progression of TAA. LncRNAs play critical roles in the regulation of cellular process such as cell growth and apoptosis (Wang et al., 2014). BRG1 was knocked down in VSMCs and a microarray was performed and the expression of HIF1A-AS1 was found to be regulated by BRG1. Overexpression of BRG1 in TAA and the interaction between *BRG1* and the lncRNA HIF1A-AS1 in VSMCs might provide clues to the molecular mechanisms involved in TAA. This should be pursued by using isolated VSMCs from TAA patients and appropriate animal models (Wang et al., 2014). The lncRNA HIF1A-AS1 plays an important role in the pathophysiology of VSMCs.

The Natural antisense (NATs) ANRIL, KCNQ1OT, and SENCR have been found expressed in VSMCs (Congrains et al., 2012). ANRIL, as discussed elsewhere in this review, also regulates the function of VSMCs. As explained below, the genomic region (9p21) coding for *ANRIL* also contains the *CDKN2A* and *CDKN2B* genes which encode the cell cycle regulators p16 (INK4a), p14 (ARF) and p15 (INK4b). The role of ANRIL in various types of aneurysms needs to be investigated. The 9p21 genotype had an influence on CDKN2A/CDKN2B/ANRIL expression levels in VSMCs, VSMC proliferation and VSMC content in atherosclerotic plaques. Analyses of VSMC primary cultures showed that the 9p21 risk genotype was associated with reduced expression of p16 (INK4a), p15 (INK4b) and ANRIL and with increased VSMC proliferation (Congrains et al., 2012).

RNA sequencing (RNA-seq) by Bell et al. on the human coronary artery SMCs showed 31 unannotated lncRNAs, including SENCR, which is enriched in vascular cells. SENCR is transcribed antisense from the 5′ end of the *FLI1* gene and exists as two splice variants (Bell et al., 2014). SENCR has a cytoplasmic location (Bell et al., 2014). RNA-seq experiments in VSMCs after SENCR knockdown revealed decreased expression of myocardin and numerous smooth muscle contractile genes, while several promigratory genes were increased. Loss-of-function studies in scratch wound and Boyden chamber assays suggests SENCR as an inhibitor of VSMC migration (Bell et al., 2014).

Finally, several lncRNAs (Lnc Ang362, Lnc-Ang 162, LncAng 112, LncAng249, and LncANg219) were found to be expressionally regulated by Ang II in VSMCs (Leung et al., 2013). The lncRNA AngII362 also mediates the proliferation of VSMC by acting on the miR221/222 (Leung et al., 2013).

### *In vivo* aneurysm models

In order to develop knowledge of ncRNAs that can be translated to clinical applications, it is important to devise “reproducible” models mimicking the features of human aneurysms, such as lesion in the elastic layer, inflammatory infiltrate within the adventitial and medial layers, and increased proteolytic activity within the aneurysm wall. Clinical relevant models of AA are still lacking, particularly for TAA. Consequently, there is large scope for improvement, as discussed below.

One of the first animal models employed for aneurysm studies is the blotchy mouse (Andrews et al., 1975). These mice have a mutation (*blotchy* allele) in the *Mottled* locus gene of the X chromosome, which leads to cross-linking abnormalities in elastin and collagen. These mice have a variety of connective tissue abnormalities, including spontaneous thoracic aneurysm formation and reduce tensile strength of the skin (Andrews et al., 1975). Aneurysms in this model are histopathologically similar to that observed in humans, with elastic fiber fragmentation and inflammatory infiltrate within the medial and adventitial layers (Andrews et al., 1975). To the best of our knowledge, this mouse model has not been employed for ncRNA studies. By contrast, another genetic model, the *Fibulin-4(R/R*) mice have been used for studying miR-29 expression by Boon et al. (2011). Fibulin proteins form bridges that stabilize the organization of extracellular matrix structures (Hanada et al., 2007). Hanada et al. have shown that reduced Fibulin-4 expression leads to dilatation of the ascending aorta, aneurysm formation, dissection of the aortic wall and thickened aortic valvular leaflets that are associated with aortic valve stenosis and insufficiency (Hanada et al., 2007). At the expressional level, the TGF-beta signaling appears perturbed (Hanada et al., 2007). Taken together, these pathological and expressional features suggest the relevance of *Fibulin-4(R/R*) for aneurysmal studies.

Mice with an homozygous Apolipoprotein E gene depletion (*ApoE KO*) are also predisposed to elastic fiber fragmentation as well as to atherosclerosis and atherosclerotic plaque formation (Anidjar et al., 1990). In addition to these genetic models, the delivery of exogenous substances has also been employed to trigger AAA in mice. Examples are the perivascular delivery of calcium chloride or proteases (collagenases and elastases), transient perfusion of elastase into infrarenal aorta, and prolonged systemic Ang II infusion (Daugherty and Cassis, 2004). These interventions can have been performed either in wild type mice or in atherosclerotic *ApoE KO* (Azuma et al., 2009).

One study investigated the role on miR-21 and miR-29 in mice with AAA (Maegdefessel et al., 2014a). They noted that miR-21 was associated with protective qualities against AAA development. They took antagomiR against miR-21 and lentiviral vector (LV) of pre-miR-21, which would down-regulate and up-regulate miR-21, respectively. With the antagomiR-21, they noted a marked increase in the development of AAA in murine models, whereas LV-mediated miR-21 overexpression significantly decreases AAA progression. Interestingly, they also noted that in samples that were exposed to high levels of nicotine to simulate the modifiable risk factor of smoking, there was a marked increase in miR-21, and eluded to the fact that this may be a “safety mechanism,” in order to try and combat the negative effects of smoking (Maegdefessel et al., 2014a). In a different studies, The miR-21 was shown to prevent abdominal aortic aneurysms development by targeting PTEN (phosphatase and tensin homolog) (Ji et al., 2007). MiR-21 regulates growth and survival of VSMCs by decreasing the expression of PTEN and inducing expression of Bcl-2, resulting in pro-proliferative and anti-apoptotic effects in a carotid injury model in rats. In regards to aortic dilatation, miR-21 was significantly up-regulated in two established murine models of AAA disease. Among the miR-21 target genes that alter proliferation and apoptosis, PTEN was the only target gene to be significantly down-regulated at three different time points during aneurysm development and progression. miR-21 also targets multiple members of the dedicator of cytokinesis (DOCK) superfamily and modulates the activity of ras-related C3 botulinum toxin substrate 1 (Rac1) small GTPase that regulates VSMC phenotype (Ji et al., 2007).

The role of miR-29 in aneurysm formation and development of aortic dilation was confirmed by Maegdefessel et al. (2012). MiR-29 is known to be involved in the regulation of ECM within the vascular wall. Consequently, increased miR-29 expression would down-regulate ECM synthesis. In aneurismal formation, an increase in ECM is a protective quality, an attempt to increase the tensile strength of the vessel in order to prevent diameter expansion. The miR-29 family, which targets numerous gene transcripts that encode ECM proteins, is known to modulate gene expression during development and aging of the abdominal aorta and during the progression of aortic aneurysms (Boon et al., 2011; Maegdefessel et al., 2012, 2014a). Maegdefessel et al. (2012, 2014a) looked in particular at miR-29b, one of the family members of miR-29. In their murine models, they noted that there was an increase in ECM production when they inhibited miR-29b expression in their antimiR-29 mouse population. In addition to this, they also noted a concurrent decrease in MMP2 and MMP9. These findings were also seen in their human aneurysm/non-aneurysm tissue studied. This suggests a strong link with increased miR-29b expression and aneurismal development (Maegdefessel et al., 2014a). Interestingly, similar findings were also found by another team: Boon et al. (2011) looked at miR-29b in association with aneurysm formation and increasing age. Age is a significant risk factor for the development of AAA disease. They noted that vascular wall changes due to age are crucial to the development of AAA disease. In their study they found increased levels of miR-29 in aging vessel walls. Knowing that miR-29 is associated with reduced ECM production and therefore increased AAA development, they inferred from their results that the age related risks associated with AAA development were due to increased miR-29 levels and reduced wall structural integrity (Boon et al., 2011). Inflammation is another key element in the AAA development and disease progression as it has strong correlations with vessel reconstruction and alteration (Shimizu et al., 2006). One study noted that miRNA expression may be linked to inflammatory responses within vasculature, in turn, affecting AAA development (Maegdefessel et al., 2014b). They looked at miR-23b-24-27b cluster in murine models, and attempted to find a correlation with up-regulated inflammatory mediators. They found that miR-24 had the most significant negative correlation with inflammatory mediators, looking in particular at chitinas 3-like1 (CH3L1). CH3L1 is a known mediator of inflammation and has been suggested as a novel biomarker of chronic inflammatory disease (Lee et al., 2011). They found that in murine AAA models miR-24 levels are inversely correlated with increased levels of CH3L1. Inferring from these results, we can speculate that miR-24 not only has protective qualities against the inflammatory processes involved in AAA disease, but it may also be used as a biomarker for the progression of the disease. Nonetheless, there were no significant changes in circulating miR-24 between small and large AAA. However, CH3L1 levels were significantly increased in large AAA compared with small, and that using both in conjunction may be a viable biomarker of disease progression (Maegdefessel et al., 2014b). These results were noted to correlate to those in *ex-vivo* human AAA tissue.

Finally, miR-712 is the murine homolog of the aforementioned MMP activator miR-205 (Kim et al., 2014). In addition, miR-712 was proved involved with aneurysm formation in mice (Kim et al., 2014).

Concerning lncRNAs, the aforementioned Lnc Ang362, Lnc-Ang 162, LncAng 112, LncAng249, and LncANg219 were found to have altered expression altered in the aorta of Ang II treated mice (Leung et al., 2013).

Research has timidly expanded to large animal models, which better mimic the human anatomy and allow for diagnostic (including *in vivo* imaging) and therapeutic approaches similar to what could be delivered to human patients. One study in particular looked at the use of porcine arterial tissues that have been *in vivo* manipulated with a mixture of collagenases and calcium chloride in order to replicate TAA as close as possible to the ones observed in patients (Eckhouse et al., 2013). Eckhouse et al., reproduced this model and demonstrated that under the conditions they used the outcome was predictable and consistent. In addition to this, they cited the importance of finding a large mammalian model that will accurately mimic human pathology. They propose that murine models previously used in TAA studies are only useful at the cellular level, as this data is not easily transferable to human models. We concur with this opinion. Their porcine model, on the other hand, was noted to have the mural structure and contents that are seen in human TAA tissue studies, i.e., cell contents, wall constituent configuration and protease levels (in particular MMPs). The importance of this study is that the development of a reproducible TAA model that can be used in any study, providing a level of standardization and transferability to the disease in humans (Riches et al., 2013). We are currently developing the model to be used in our own translational research projects.

### *Ex-vivo* aneurysm models

A recent study co-authored by one (TGA) of us, used porcine arterial segments to create an *ex-vivo* model of aneurysm comparable to human AAA. Freshly isolated porcine arteries were pretreated with collagenase and elastase before being cultured under flow in a bioreactor for 12 days. This produced aneurysmal-like remodeling. VSMCs were harvested from the porcine aneurysmal vessels and further studied *in vitro* in comparisons with cells prepared from human end stage AAA disease. Cellular senescence was investigated using β-galactosidase staining and apoptosis was quantified using a fluorescence-based caspase 3 assay. The data on VSMC morphology, senescence and proliferation were comparable to that of end stage human AAA vessels (Riches et al., 2013). The authors propose this model can be used to examine temporal changes in VSMC biology in porcine and also human aortic vessels and to identify the molecular mechanisms conductive to or protective from aneurysmal remodeling (Riches et al., 2013). In the longer term, this may inform and functionally validate new therapeutic targets (including miRNAs and lncRNAs) as part of a translational pipeline preceding human interventional studies.

### Work on clinical samples and patient population

In recent years miRNAs have been found to regulate vascular pathologies in aortic aneurysm (thoracic and abdominal disease).

#### Expressional studies

Liao et al. performed microarray and systemic analysis of miRNA from the ascending aorta segments of TAA patients and normal aorta from organ donors (Liao et al., 2011). The resulting miRNAs were validated by qPCR and target genes were predicted TargetScan v5.1 and Mirind V5.1. The miRNAs which were validated as significantly upregulated in human TAA are miR-183^*^, −433, −553, −491-3p, −30c, and −338-5p. By contrast, miR-143, −145, −22, −24, −93 and −768-5p were lower in the TAA samples (Liao et al., 2011). Elia et al. also found that miR143 and 145 are down regulated in ascending aortic aneurysms (Elia et al., 2009). Loss of miR-143 and miR-145 expression induces structural modification of the aorta due to an incomplete differentiation of VSMC. This confirms a previous study which showed transition of VSMC from contractile to synthetic phenotype exists in media of TAA aorta, indicating that VSMC are less differentiated in TAA (Elia et al., 2009).

In another study, Jones et al. compared miRNA expression levels in aortic tissue specimens collected from patients with ascending TAAs and looked at the aneurysm progression (Jones et al., 2011). They observed decreased expression of miRs −1, −21, −29a, −133a, and −486 as compared with normal aortic specimens. A significant relationship between expression of miR-1, −21, −29a, and −133a and aortic diameter was identified; as aortic diameter increased, miR expression decreased (Jones et al., 2011). Through the use of a bioinformatics approach, members of the matrix metalloproteinase (MMP) family, proteins involved in TAA development, were examined for putative miR binding sites. MMP2 and MMP9 were identified as potential targets for miR-29a and miR-133a, respectively, and MMP-2 was subsequently verified as a miR-29a target *in vitro* (Jones et al., 2011). Reduced miR29-b was also replicable in human aneurysmal tissue samples compared with controls (Maegdefessel et al., 2012).

Kumari et al. compared ascending TAA with descending TAA tissue using miRNA microarray and found that the expression level of miR-1, 30c-2^*^, miR-145, miR-204, miR-331-3p were up-regulated in ascending TAA in comparison with descending TAA (Premakumari et al., 2014). Importantly this data suggests that these miR mayhave biological and clinical relevance in the context of TAAs and may provide significant targets for diagnostic/prognostic and therapeutic applications (Premakumari et al., 2014).

Pahl et al. compared miRNA expression levels using an affymetrix GeneChip miRNA array in human AAA vs. control human aorta tissue (Torella et al., 2011). Three miRNA were found upregulated in AAA: miR 181a^*^, miR 146a, miR21. While five miRNA miR 133b, miR 133a, miR 331-3p, miR 30c-2^*^, and miR204 were significantly down regulated in human AAA tissue. The down regulated miRs replicated well in the qPCR validation while the upregulated three miRs failed to replicate. In addition, they found that miR 133 is down regulated in human AAA tissue (Torella et al., 2011).

LncRNA expression has not yet been investigated in clinical samples. However, we expect that this gap will be soon filled, including by our group. Because of the high number of lncRNAs, next generation sequencing methods will be useful in developing these studies.

#### Genetic studies

Genome-wide association studies (GWAS) is an approach used to identify associations between single nucleotide polymorphisms (SNP), variations in a single nucleotide within the DNA sequence, and their influence on health and disease. AA is a complex disease with important risk factors like male sex, cigarette smoking, a personal history of myocardial infarction and a heritability of 70% i.e., family history (Wahlgren et al., 2010). GWAS suggest a role of ANRIL in AA. There are five chromosomal regions in the human genome with the strongest supporting evidence of contribution to the genetic risk for AAA: (1) *ANRIL* (also known as *CDKN2BAS*) on chr9p221 locus is expressed in humans. 9p21.3. This locus is among the most strongly replicated regions for cardiovascular disease and is also associated with coronary artery disease. *ANRIL* encodes an antisense lncRNA that regulates expression of the cyclin-dependent kinase inhibitors *CDKN2A* and *CDKN2B;* (Congrains et al., 2012) (2) DAB2 interacting protein (*DAB2IP*), which encodes an inhibitor of cell growth and survival; (3) low density lipoprotein receptor-related protein 1 (*LRP1*), a plasma membrane receptor involved in VSMC and macrophage endocytosis; (4) low density lipoprotein receptor (*LRPR*); and (5) contactin-3 (*CNTN3*) (Boucher et al., 2003; Elmore et al., 2009; Gretarsdottir et al., 2010; Bown et al., 2011).

Candidate gene studies represent complementary approaches to analyze genetics behind AA. The most significant associations with AAA discovered in candidate gene studies are: (1) *SORT1* (lipid metabolism) (Jones et al., 2013); (2) *IL6R* (inflammation) (Harrison et al., 2013); (3) *LPA* (lipid metabolism); (4) *AGTR1* (Renin angistenin system) (Jones et al., 2008); (5) *TGFBR2* (TGFB signaling) (Biros et al., 2011); (6) *MMP3* (Degradation of ECM) (Yoon et al., 1999); (7) *MTHFD1* (methionine metabolism) (Hinterseher et al., 2011); (8) *LRP5* (lipid metabolism) (Boucher et al., 2003). To date, genetic studies targeting ncRNAs are lacking.

## Summary and future research

Today, there are no clinical tests to detect an aneurysm and the management of aneurismal diseases is unsatisfactory. Surgical intervention is the only valuable treatment option and based only on size. Predictors of biological behavior of an aneurysm wall would enhance the management of aneurysm disease from a one dimensional surgical strategy. This strong clinical need has prompted the research of new biomarker and therapeutic targets to be to translate into clinical care. The explosion on miRNA research in the last year delivered some candidates to be further studied. Basic science still needs to fill several gaps. Firstly, studies in ncRNA biology have focused on the effects of miRNAs or lncRNAs on VSMC and ECs. This research needs to be extended to include miRNAs or lncRNAs in other cellular components of blood vessels, such as fibroblasts and macrophage, which remain to be investigated. NcRNA research focused on their function in blood vessels is currently limited. In addition to miRNAs and lncRNAs, other ncRNAs, such as rasiRNAs and piRNAs, may also be involved in the development of cardiovascular pathology, and their function requires evaluation. Additional work on miRNA drug chemistry and delivery approaches enabling local and systemic but safe delivery of ncRNA-based therapeutics is required, this further necessitates the use of clinical relevant animal models and *in vivo* imaging systems. lncRNAs are vastly unexplored by cardiovascular researchers, but given the enthusiasm of many academic and industry-based scientists who specialize in ncRNAs research, we foresee this will be reversed by a plethora of exciting studies.

### Conflict of interest statement

The authors declare that the research was conducted in the absence of any commercial or financial relationships that could be construed as a potential conflict of interest.

## Abbreviations

AA: arterial aneurysm
AAA: abdominal aorta aneurysm
Ang II: angiotensin II
ANRIL: antisense non-coding RNA in the INK4 locus
ARF: alternate reading frame
BAV: bicuspid aortic valve
BRG: Brahma-related gene
DOCK: dedicator of cytokinesis
EC: endothelial cell
ECM: extracellular matrix
ELN: elastin
ERK: extracellular-signal-regulated kinase
HIF: hypoxia-inducible factor
JNK: c-Jun NH(2)-terminal kinase
KLF: Krüppel-like factor
lncRNA: long non coding RNA
miRNA: miR, microRNA
MMP: matrix metalloproteinases
ncRNA: non coding RNA
PPE: porcine pancreatic elastase
PTEN: Phosphatase and tensin homolog
rRNA: ribosomal RNA
SMC: smooth muscle cell
SnoRNA: small nucleolar RNA
SNP: single nucleotide polymorphism
TAA: thoracic aortic aneurysm
TIMP: tissue inhibitors of metalloproteinase
TGFβ: transforming growth factor β
TNFα: tumor necrosis factor α
tRNA: transfer RNA
VSMC: vascular smooth muscle cell.
